# Water Purification and Microplastics Removal Using Magnetic Polyoxometalate‐Supported Ionic Liquid Phases (magPOM‐SILPs)

**DOI:** 10.1002/anie.201912111

**Published:** 2019-11-27

**Authors:** Archismita Misra, Christian Zambrzycki, Gabriele Kloker, Anika Kotyrba, Montaha H. Anjass, Isabel Franco Castillo, Scott G. Mitchell, Robert Güttel, Carsten Streb

**Affiliations:** ^1^ Institute of Inorganic Chemistry I Ulm University Albert-Einstein-Allee 11 89081 Ulm Germany; ^2^ Institute of Chemical Engineering Ulm University Albert-Einstein-Allee 11 89081 Ulm Germany; ^3^ Helmholtz Institute Ulm Helmholtzstrasse 11 89081 Ulm Germany; ^4^ Instituto de Ciencia de Materiales de Aragón (ICMA-CSIC) CSIC-Universidad de Zaragoza 50009 Zaragoza Spain

**Keywords:** ionic Liquids, microplastics, polyoxometalate, self-assembly, water purification

## Abstract

Filtration is an established water‐purification technology. However, due to low flow rates, the filtration of large volumes of water is often not practical. Herein, we report an alternative purification approach in which a magnetic nanoparticle composite is used to remove organic, inorganic, microbial, and microplastics pollutants from water. The composite is based on a polyoxometalate ionic liquid (POM‐IL) adsorbed onto magnetic microporous core–shell Fe_2_O_3_/SiO_2_ particles, giving a magnetic POM‐supported ionic liquid phase (magPOM‐SILP). Efficient, often quantitative removal of several typical surface water pollutants is reported together with facile removal of the particles using a permanent magnet. Tuning of the composite components could lead to new materials for centralized and decentralized water purification systems.

## Graphical abstract

**Clean machine**: A three‐component composite allows the magnetic removal of organic, inorganic, microbial, and microplastic pollutants from water. Magnetic Fe_2_O_3_/SiO_2_ core–shell nanoparticles are functionalized with a polyoxometalate ionic liquid. The composite can be dispersed in water, binds multiple pollutants and can be fully recovered with a permanent magnet.

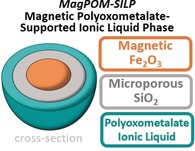

Access to clean water is still a major challenge in large parts of the world, and many water resources in developing countries carry high concentrations of organic pollutants, heavy metals or microbial contaminants.^1^, ^2^, ^3^ In addition, microplastic particles have recently been identified as contaminants of emerging concern (CEC) which can enter the food chain upon uptake by marine organisms.^4^, ^5^ Microplastics can bind and concentrate persistent organic pollutants (POPs), which amplifies their public health impact.^6^ Often, water purification relies on a series of operations including chemical coagulation, flocculation, sedimentation, filtration and disinfection which produce safe drinking water from contaminated surface or ground water.^7^, ^8^

Traditionally, different filters which target specific pollutants are connected in line to enable stepwise water purification. Filter materials typically include porous adsorbents, such as zeolites, minerals, or active carbon.^9^ However, treatment of large volumes of water or the deployment in remote areas require alternative methods which combine ease of use with minimum technological requirements and the ability to simultaneously remove multiple contaminants. Composites are promising materials to this end, as their target properties can be tuned by independent modification of each component. Recently, some of us have explored the removal of water contaminants by developing so‐called polyoxometalate‐supported ionic liquid phases (POM‐SILPs).^10^ This composite is based on commercial porous silica particles which are surface‐functionalized with water‐immiscible polyoxometalate ionic‐liquids (POM‐ILs) capable of binding organic and inorganic contaminants.^11^ The POM‐ILs^12^ combined lacunary Keggin tungstate anions featuring heavy‐metal binding sites^13^, ^14^ with long‐chain quaternary organo‐ammonium cations^15^, ^16^ which act as antimicrobials. Integration of the POM‐SILP composite in filter cartridges allowed the simultaneous removal of organic, inorganic, and microbial contamination from water. However, the system requires filtration processing which is limited to small water volumes, and overcoming this challenge is either energy‐intense (using pressurized systems) or materials‐intense (using more filter materials).

Herein, we propose water purification by magnetic particles as a promising alternative to filtration which could be employed in various water treatment scenarios. In contrast to filtration, magnetic water purification could facilitate the treatment of large volumes of water, and can in principle be used without further infrastructure if particle removal is possible using simple permanent magnets.^17^ Pioneering studies have explored the removal of aqueous pollutants using magnetic particles, including heavy‐metal cation removal by amino acid‐modified iron oxide,^18^ organic pollutant removal by graphene oxide‐functionalized magnetic particles,^19^ and separation of freshwater algae using silica‐coated magnetic particles.^20^ Recently, ground‐breaking studies reported the use of light‐driven magnetic microswimmers for the collection and removal of microplastics from water.^21^

Herein, we target multi‐pollutant removal based on core–shell particles composed of a superparamagnetic iron oxide (Fe_2_O_3_, hematite) core encased in a porous silica shell.^22^, ^23^ We hypothesized that this architecture enables magnetic removal and at the same time stable POM‐IL surface‐anchoring. We demonstrate that the resulting magnetic POM‐SILP (magPOM‐SILP) composite effectively binds organic, inorganic, microbial, and microplastic pollutants from water, and can easily be recovered using a permanent magnet. Treatment of large water volumes therefore becomes possible. To our knowledge, this is the first report of magnetic POM‐SILPs (MagPOM‐SILPs), therefore their synthesis is described briefly: the magnetic iron oxide/silica core–shell precursor particles are synthesized by an adapted reverse water‐in‐oil microemulsion method at elevated temperature with cyclohexane as the organic phase.^22^ Reaction of the surfactant Brij 56 with aqueous Fe^III^ solution and subsequently with Si(OEt)_4_ leads to spherical Fe_2_O_3_@SiO_2_ core–shell nanoparticles which were washed, dried, and calcined (420 °C) to give the microporous Fe_2_O_3_@SiO_2_ composite **1**. The average particle size of **1** was 16 nm, the BET specific surface area was approximately 270 m^2^ g^−1^ and the BJH pore volume was 0.97 cm^3^ g^−1^; for further characterization details, see Supporting Information. Composite **1** is used as non‐modified reference compound throughout this study.

The POM‐IL is synthesized as described in the literature^10^, ^12^, ^24^, ^25^ by combination of the lacunary‐Keggin cluster anion ([α‐SiW_11_O_39_]^8−^)^26^ and the antimicrobial tetra‐*n*‐heptyl ammonium cation (Q^7^ (=(*n*‐C_7_H_15_)_4_N^+^)^27^ (Figure 1). The magPOM‐SILPs were prepared by dispersing SiO_2_@Fe_3_O_4_ particles (4.0 g) in a POM‐IL solution in acetone (50 mL, [POM‐IL]=3.36 mm, *m*(POM‐IL=1.0 g) and subsequent vacuum drying, giving the composite magPOM‐SILP **2** with a POM‐IL loading of 20 wt %, see Supporting Information for details. As a result of the partial filling of the pores in **2** with the POM‐IL, the BET specific surface area of **2** was reduced to around 100 m^2^ g^−1^ and the BJH pore volume was 0.70 cm^3^ g^−1^; for further characterization details, see Supporting Information. Compound **2** was obtained as dry and free‐flowing powder which can be easily handled, while the POM‐IL precursor is a highly viscous liquid which would make deployment for water purification difficult.

**Figure 1:**
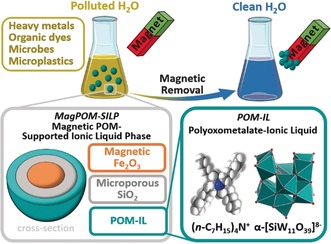
Concept of the removal of multiple pollutants from water using magnetic polyoxometalate supported ionic liquid phases (magPOM‐SILPs). Color scheme (bottom right): teal polyhedra [WO_6_], gray C, red O, blue N, white H.

We performed a series of water‐purification tests using the magPOM‐SILPs for removal of pollutants often found in water samples. Aqueous samples (5 mL) of the respective pollutant at health‐relevant concentrations were prepared, and the magPOM‐SILP **2** or the reference particles **1** (50 mg) were dispersed in the polluted sample and magnetically stirred. After stirring for 24 h, the magnetic particles were removed using a permanent magnet (see Supporting Video). Note that no leaching of any components of **2** into the aqueous phase was observed after stirring for 24 h using inductively coupled plasma atomic emission spectroscopy (ICP‐AES) and C,H,N, elemental analysis.

We explored heavy‐metal removal from water using the metal‐ion pollutant models^2^ Pb^2+^, Ni^2+^, Co^2+^, and MnO_4_^−^ according to the standard procedure described above (Figure 1). The metal‐ion concentrations are set to levels significantly above the WHO guideline levels to test removal efficiency and simulate acute pollution scenarios (Table 1).^28^ ICP‐AES analyses of the solutions after particle removal show metal removal efficiencies between 75–99 mol % for the magPOM‐SILP **2**, while the non‐modified reference **1** showed significantly lower removal efficiencies in the approximately 35–50 mol % range (Table 1, entries 1–5).

**Table 1:** Pollutant removal performance of magPOM‐SILP **2** and the non‐modified reference **1**.^[a]^

Entry	Pollutant (conc.) *[WHO guideline level]* 28	Pollutant removal by magPOM‐SILP **2** *[non‐modified reference* **1** *]* ^[a]^
1	Pb^2+^ (2.2 mm) *[0.05 μM]*	99 *[48]* mol %
2	Ni^2+^ (1.3 mm) *[0.34 μM]*	90 *[52]* mol %
3	Cu^2+^ (1.3 mm) *[30 mM]*	99 *[51]* mol %
4	Co^2+^ (1.3 mm) *[–]*	75 *[35]* mol %
5	MnO_4_^−^ (1.15 mm) *[0.05 μM]*	99 *[42]* mol %
6	PBV (32 μm)^[b]^ *[–]*	99 *[6]* mol %
7	PS beads, 10 μm (1 g L^−1^) *[–]*	100 *[0]* wt %
8	PS beads, 1 μm (1 g L^−1^) *[–]*	100 *[0]* wt %

[a] *V*_solution_=5 mL; *m*_adsorbent_ (**1** or **2**)=50 mg, *t*_binding_=24 h. [b] Patent Blue V.

We then explored the removal of organic pollutants from water using the triphenylmethane (trityl) dye Patent Blue V (PBV, Figure 2) as a model for textile dye pollutants.^29^ To this end, aqueous solutions of PBV were stirred with magPOM‐SILP **2** using the standard experimental procedure described above. Dye removal was quantified by UV/Vis spectroscopy (Figure 2) and it was observed that the magPOM‐SILP **2** removes more than 99 % of the dye while the non‐modified reference **1** showed only 6 % removal (Table 1, entry 6). The increased dye removal by **2** is assigned to the high affinity of the POM‐IL to interact with organic species, due to the large, hydrophobic Q^7^ cations.^11^

**Figure 2:**
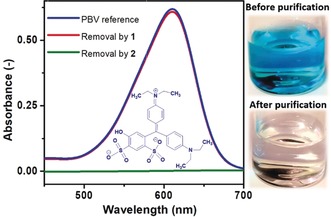
Removal of the water‐soluble aromatic model pollutant Patent Blue V (PBV) from water. Left: UV/Vis spectra before purification (blue), after purification using reference **1** (red) and after purification using magPOM‐SILP **2** (green). [PBV]_0_=32 μm. Adsorption time: 24 h. Details see Table 1 entry 6. Inset: molecular structure of PBV. Right: photographs of the PBV solutions before and after purification (with **2**).

We hypothesized that the viscous POM‐IL coating on the magnetic nanoparticle surface could be well suited for attaching the magPOM‐SILP particles to microplastics, and thereby enable their magnetic recovery from water. To this end, we used commercial colloidal solutions of spherical polystyrene (PS) beads (diameter 1 μm and 10 μm, PS bead concentration: 0.1 wt % (=1 g L^−1^)) as models of environmentally persistent microplastics. PS particle removal was quantified using dynamic light scattering (DLS, see Supporting Information for details). The removal experiments were carried as described above (*V*_solution_=5 mL, *t*_binding_=24 h). Our experiments demonstrated quantitative removal of both the 1 μm and 10 μm PS beads using magPOM‐SILP **2**. In contrast, the reference **1** showed no microplastics removal, see Table 1, entries 7 and 8.

To gain some insights into the kinetics of the binding of **2** to the microplastic particles, we performed the removal experiment described above (using both PS bead sizes) at a reduced binding time of 6 h, which also resulted in quantitative PS bead removal. In addition, we demonstrated that three consecutive recycling runs using the same batch of **2** are possible, all of which show quantitative removal of both the 1 μm and the 10 μm PS beads (see Supporting Information). Next, we examined the microplastics removal capacity of **2** by performing the above experiment, but using PS bead solution volumes of 20 mL and 50 mL. For both solutions we note PS bead removal efficiencies over 90 % based on DLS analyses, see Supporting Information. This emphasizes that the magPOM‐SILPs are capable of removing microplastic model compounds from large volumes of water.

To gain insights into the interactions between magPOM‐SILP **2** and the PS beads, we performed scanning electron microscopy/energy‐dispersive X‐ray spectroscopy (SEM/EDX) of the magnetically recovered, dried samples. As shown in Figure 3, the significantly smaller particles of **2** cover large parts of the surface of the PS beads and thus render them susceptible for magnetic removal. We suggest that this surface attachment of **2** to the PS beads is due to hydrophobic interactions between the POM‐IL coating and the PS surface, see Supporting Information for proposed scheme. In addition, PS bead aggregation is observed which could be induced by **2**, and further aggregation studies are currently underway to understand the surface attachment in more detail. In sum, PS microplastics removal by magnetic particle attachment could provide a means of treating larger volumes of water which are not amenable to classical filtration.^21^

**Figure 3:**
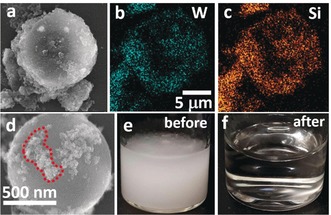
Microplastics removal by magPOM‐SILP **2**: a)–c): SEM and EDX elemental mapping micrographs showing 10 μm polystyrene beads covered with **2** (as indicated by the W and Si signals). d) high magnification micrograph of a 1 μm polystyrene sphere coated with a smaller loading of **2** (outlined in red). e),f) photographs of test solutions before and after microplastics removal.

Some of our previous research has confirmed the bactericidal properties of POM‐ILs,^10^, ^12^, ^25^ therefore we hypothesized that magPOM‐SILP **2** would be able to purify water heavily contaminated with bacteria. The antibacterial water purification properties of the magPOM‐SILPs were tested against gram‐negative *E. coli* and gram‐positive *B. subtilis*.^12^ Briefly, aqueous solutions of the microporous magPOM‐SILP **2** were inoculated with 10^6^ CFU mL^−1^ of *E. coli* or *B. subtilis* and incubated at 37 °C for 1 h before removing the particles with a magnet and quantifying the bacteria present in the supernatant solutions. At a magPOM‐SILP **2** concentration of 1 mg mL^−1^, the bacterial removal was 58 % for *E. coli* and 100 % for *B. subtilis*, while at a concentration of 10 mg mL^−1^ the bacterial removal efficiency was 100 % for both bacterial strains. The antibacterial effect was confirmed and characterized using electron microscopies (SEM and TEM). Figure 4 B and Figures S3–S6 illustrate how the morphology of the bacteria was affected by the presence of magPOM‐SILP **2**, even at concentrations below the minimum bactericidal concentration.

**Figure 4:**
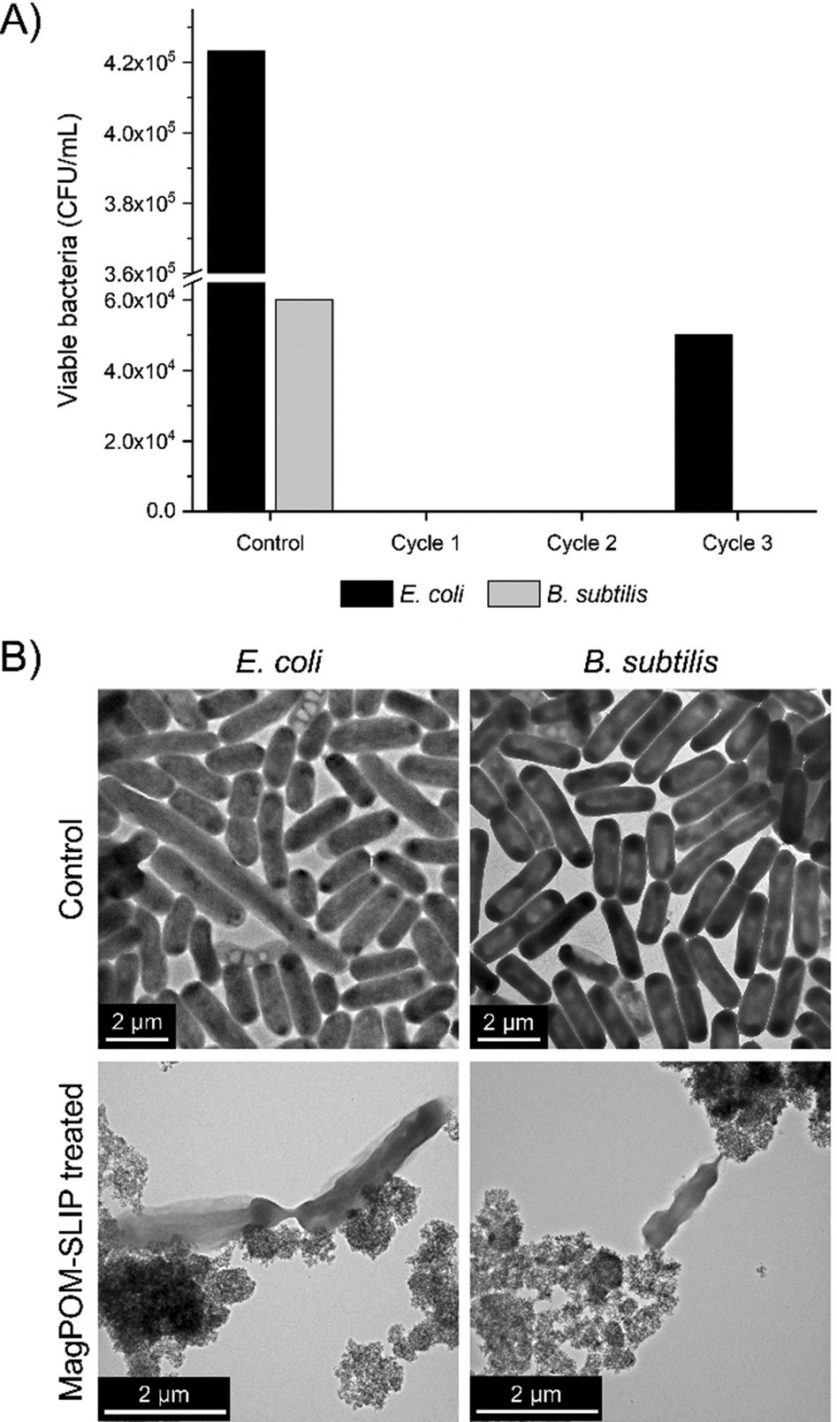
A) bacteria removal efficiency of magPOM‐SILP **2** (10 mg mL^−1^) over three consecutive cycles (50 min/cycle), where the initial concentration of the *E. coli* and *B. subtilis* inoculum was 10^6^ CFU mL^−1^ (37 °C, pH 6); B) TEM images of *E. coli* and *B. subtilis* incubated with magPOM‐SILP **2** at sub‐bactericidal concentrations, including control bacterial cells without magPOM‐SILP **2**.

The reusability of the magPOM‐SLIP nanoparticles was tested over three cycles of inoculation with *E. coli* or *B. subtilis*, particle separation, and particle washing with water and subsequent reuse with a fresh inoculum of the bacteria. The bactericidal effect of **2** against *B. subtilis* remained unaltered after three cycles, while the effect of the particles on *E. coli* was reduced after the second cycle (Figure 4 A), which is commensurate with other studies using magnetic nanoparticles for water purification.^30^, ^31^

In conclusion, we report the first example of magnetic polyoxometalate‐supported ionic liquid phases (magPOM‐SILPs) and their use in water purification. The magPOM‐SILP composite is capable of removing organic, inorganic, microbial, and microplastic pollutants from water using a range of target‐specific removal modes. High removal efficiencies are reported together with initial insights into a new mode of microplastics removal by surface‐binding of magnetic particles. In future, we will explore how optimization of the individual components can be used to improve the capacity of the systems and investigate their coupling to electromagnetic recovery systems for use under more realistic operating conditions.

## Conflict of interest

The authors declare no conflict of interest.

## Supporting information

As a service to our authors and readers, this journal provides supporting information supplied by the authors. Such materials are peer reviewed and may be re‐organized for online delivery, but are not copy‐edited or typeset. Technical support issues arising from supporting information (other than missing files) should be addressed to the authors.

Supplementary

Supplementary
