# Supplementary material for: Water Purification and Microplastics Removal Using Magnetic Polyoxometalate‐Supported Ionic Liquid Phases (magPOM‐SILPs)
Source: Angew Chem Int Ed Engl. 2019 Nov 27;59(4):1601–5. doi: 10.1002/anie.201912111 (PMC7004052; doi:10.1002/anie.201912111)
Supplement: Supplementary file 1 — Supplementary [file ANIE-59-1601-s001.pdf]

Angewandte Chemie
Eine Zeitschrift der Gesellschaft Deutscher Chemiker

Supporting Information

Water Purification and Microplastics Removal using Magnetic Polyoxometalate-Supported Ionic Liquid Phases (magPOM-SILPs)

Archismita Misra, Christian Zambrzycki, Gabriele Kloker, Anika Kotyrba, Montaha H. Anjass, Isabel Franco Castillo, Scott G. Mitchell, Robert Güttel,* and Carsten Streb**

anie_201912111_sm_miscellaneous_information.pdf
anie_201912111_sm_Movie_s.mp4

Supporting Information

Table of Contents

1. Instrumentation
2. Synthesis and characterization
3. Experimental methodology and analysis
4. Antibacterial activity assays
5. References
6. Author Contributions

SUPPORTING INFORMATION

1. Instrumentation

Elemental Analysis: Elemental analysis (CHN) was performed on a *Euro Vector Euro EA 3000 Elemental Analyzer*.

FT-IR Spectroscopy: FT-IR spectroscopy was performed on a *Shimadzu FT-IR 8400* spectrometer. Samples were prepared as KBr pellets. Signals are given as wavenumbers in cm^{-1} using the following abbreviations: *vs* - very strong, *s* - strong, *m* - medium, *w* - weak and *b* - broad.

Inductively coupled plasma atomic emission spectrometry (ICP-AES): ICP-AES was performed on a *Perkin Elmer Plasma 400 spectrometer.*

UV-Vis Spectroscopy: UV-Vis spectroscopy was performed on a Shimadzu UV-2450 spectrophotometer, Varian Cary 50 spectrophotometer or Varian Cary 5G spectrophotometer. All systems were used with standard cuvettes (d = 10.0 mm).

Scanning Electron Microscopy (SEM) and Energy Dispersive X-ray Spectroscopy (EDX): SEM and EDX analytical data were obtained from a *Hitachi 5200 SEM* or a Zeiss Scanning Electron Microscope Leo 1550 VP equipped with an Oxford Instruments EDX system. Bacteria were analysed using a field emission SEM Inspect F50 with an EDX system INCA PentaFETx3 (FEI Company, Eindhoven, The Netherlands) in the energy range 0-30 keV.

Transmission Electron Microscopy (TEM): Bright field TEM micrographs were taken by a *Zeiss TEM 109* equipped with a CCD camera. Bacteria were analysed using a TECNAI T20 electron microscope (FEI) working at 60 kV.

Dynamic Light Scattering (DLS): DLS measurements were carried out on a *Malvern Zetasizer Nano-ZS*.

Nitrogen Physisorption: Sample degassing was performed on a *Micromeritics Smart VacPrep* at 100 °C for 12h. Nitrogen sorption isotherms were acquired on a *Micromeritics 3Flex* at T = 77 K and evaluated using Micromeritics 3Flex Software Version 5.00. N_2 was used as adsorbate. Calculation of the BET surface area was accomplished by the bet_autofit_v0712 method from Micromeritics. The evaluation of the pore distribution after BJH was done by using Harkins and Jura thickness curve and the Kruk-Jaroniec-Sayari correction. The evaluation of the micropores was realized by use of a adapted Harwarth-Kawazoe method estimating a cylindrical pore geometry after Saito-Foley.

Antibacterial Assays: All experiments were carried out under sterile conditions in a Class I Biosafety Cabinet. All data are based on six replicas to obtain average values and standard deviations and each experiment was repeated a minimum of three separate times to verify the results between different inoculations. *Escherichia coli* DH5-alpha as Gram-negative (G-) bacteria and *Bacillus subtilis* 1904-E as Gram-positive (G+) bacteria were used in the assays. Luria-Bertani (LB) liquid medium (Miller's formulation) and Nutrient Broth (NB) liquid medium were freshly prepared and sterilized by autoclave. Trypticase Soy Agar (TSA) plates were purchased from Thermo Scientific ™.

General Remarks: All chemicals were of reagent grade and purchased from SIGMA ALDRICH, ABCR CHEMICALS or ACROS ORGANICS. The chemicals were used without further purification and the reactions were carried out under ambient atmosphere, unless stated otherwise.

2. Synthesis and characterization

2.1 Synthesis of $K_8[\alpha\text{-}SiW_{11}O_{39}].13H_2O$

The lacunary Keggin POM was synthesized and characterized following an established literature procedure.[1–4]
ICP-AES for $K_8[\alpha\text{-}SiW_{11}O_{39}].13H_2O$ in wt. - % (calculated values within brackets): K 9.70 (9.71), Si 0.87 (0.87), W 61.94 (62.78).
FT-IR (characteristic bands in cm^{-1}): 3418 (s), 2367 (w), 1611 (m), 1117 (w), 996 (m), 961 (s), 890 (s), 797 (s), 727 (s), 530 (m), 508 (m), 471 (m).

2.2 Synthesis of $(Q^7)_8[\alpha\text{-}SiW_{11}O_{39}]$

The POM-IL was synthesized and characterized following a reported procedure.[2,4]
Elemental analysis for $(Q^7)_8[\alpha\text{-}SiW_{11}O_{39}]$ in wt. - % (calculated values in brackets): C 44.27 (45.14), H 7.71 (8.12), N 2.08 (1.88).
FT-IR (characteristic bands in cm^{-1}): 2960/ 2928/ 2863 (s), 1680 (w), 1656 (w), 1480/ 1463 (m), 1374 (m), 1239 (w), 1146 (w), 1068/1050 (w), 980 (m), 940 (m), 887 (s), 796 (s), 726 (s), 540 (m), 520 (w), 461 (w).

2.3. Synthesis of the magnetic iron oxide / silica core-shell particles $Fe_2O_3@SiO_2$ (composite 1)

Spherical silica-encapsulated iron oxide (Fe_2O_3) core-shell particles were prepared using an adapted reverse microemulsion method.[5] For the oil phase cyclohexane (100 mL, VWR Chemicals, AnalaR NORMAPUR®) was mixed with polyoxyethylene (10) cetyl ether (34.2 g, Brij® C10, Sigma Aldrich/Merck) in a round-bottom flask and heated to 50 °C. Subsequently, aqueous $FeCl_3$ x 6 H_2O solution (5 mL, 5 mM, ACROS Organics, p.a.) was added to the mixture under continuous stirring. After 30 min of homogenization hydrazine hydrate (3 mL, aqueous solution, 35 wt%, Sigma Aldrich/Merck) was added dropwise and slowly to the solution followed by continuously stirring for 1 h. The colour of the solution changed from bright yellow to dark brown, which indicates the formation of iron oxide nanoparticles. To encapsulate the obtained iron oxide nanoparticles in silica, tetraethyl orthosilicate (21.4 mL, TEOS, Merck, p.a.) was added to the suspension followed by aqueous ammonia solution (3 mL, 5 M, Carl Roth, 30 wt%, ROTIPURAN®). This mixture was stirred continuously for 2 h. Afterwards the microemulsion was destabilized by addition of 2-propanol (10 mL, VWR Chemicals, technical) and centrifuged at 8000 rpm for 15 min. The obtained particles were washed several times in 2-propanol, dried over night at 80°C and calcined at 420°C for 4 h (heating ramp 2 K/min) under air, giving composite **1** in yields of 30 %.

2.4. Synthesis of magPOM-SILP (composite 2)

$(Q^7)_8[\alpha\text{-}SiW_{11}O_{39}]$ (0.17 mmol, 1.00 g) was dissolved in acetone (50 mL) to give a 3.36 mM POM-IL solution. Next, composite **1** (4.01 g,) was dispersed in the POM-IL solution and the dispersion was gently shaken for 5 minutes. The solvent was then removed under reduced pressure. Acetone (50 mL) was added, and the shaking/solvent removal procedure was repeated 3-5 times. After vacuum-drying, the pure composite **2** (POM-IL loading: 20 wt-%) was obtained as a solid, free-flowing brown powder. The POM-IL loading corresponds to 20 wt.-%. Yield: 5.00 g, quantitative.

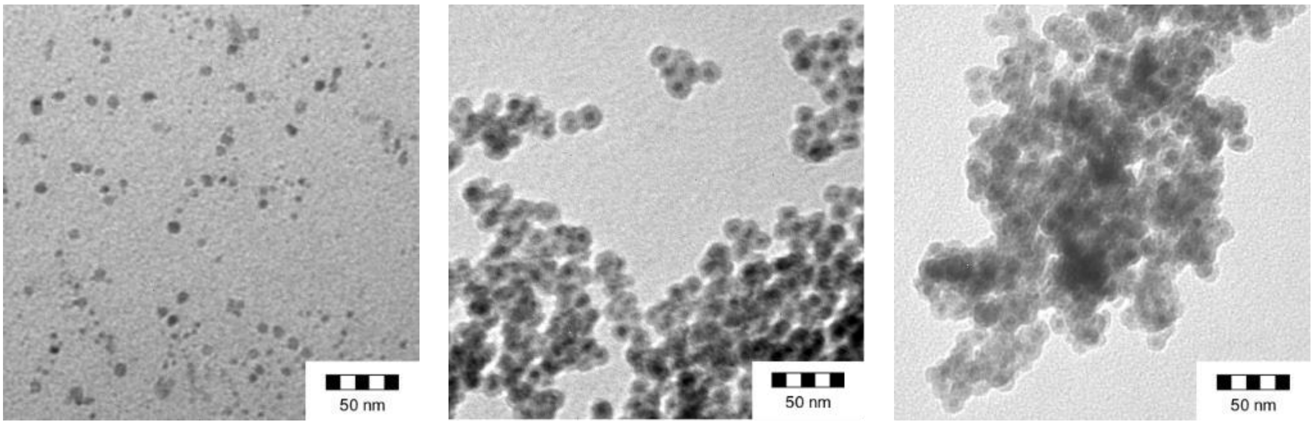

Figure S1: TEM micrographs of iron oxide nanoparticles (left); SiO_2 encapsulated iron oxide particles after calcination ($Fe_2O_3@SiO_2$: composite **1**, center); POM-IL-functionalized core-shell particles (magPOM-SILP: composite **2**, right).

2.5 Nitrogen physisorption analysis of 1 and 2

Nitrogen physisorption was used to investigate the changes of porosity after modification of the reference composite **1** with POM-IL to give the magPOM-SILP **2** (POM-IL loading: 20 wt%). Upon POM-IL binding, the specific surface area and the pore volume are decreased. This indicates that the POM-IL is bound on the internal and external surface of the microporous core-shell particles, details see Table S1.

Table S1. Nitrogen sorption results for non-modified composite **1** and magPOM-SILP **2**

	**magPOM-SILP 2**	**Reference Composite 1**
**Pore–width (Harwardt-Karwazoe)**	8.722 Å	8.701 Å
**BET surface area**	101.52 m²/g	266.44 m²/g
**BJH pore area adsorption**	125.23 m²/g	227.39 m²/g
**BJH pore area desorption**	115.50 m²/g	213.65 m²/g
**BJH pore volume adsorption**	0.6956 cm³/g	0.9717 cm³/g
**BJH pore volume desorption**	0.6907 cm³/g	0.9637 cm³/g

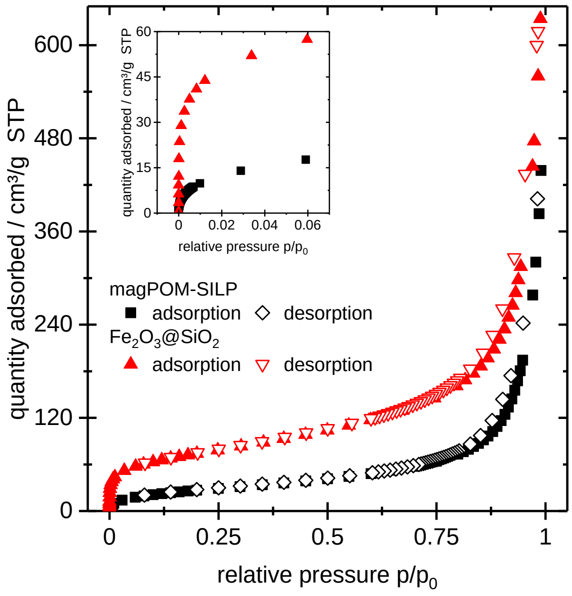

Figure S2: Nitrogen sorption isotherms of composite **1**: Fe_2O_3@SiO_2 and composite **2**: magPOM-SILP at T = 77 K. Inset shows the micropore region in detail.

3. Water purification tests and analyses

3.1 Magnetic removal of organic and inorganic pollutants

Aqueous samples (5 mL) of the respective pollutant at health-relevant concentrations were prepared, and the magPOM-SILP **2** (50 mg) or the reference composite **1** (50 mg) were dispersed in the polluted sample and magnetically stirred. After stirring for 24 h, the magnetic particles were removed using a permanent magnet. The organic dye Patent Blue V (PBV) was used as the model organic pollutant. The following metal salts were used as the source for the respective heavy metal cations or metal oxoanions: $PbNO_3$; $NiCl_2$. 6 H_2O; $CuSO_4$. 5 H_2O; $CoCl_2$. 6 H_2O and $KMnO_4$. Aqueous solutions of the respective salt were prepared at the concentrations given in Table 1, main manuscript. The extent of pollutant removal was evaluated by UV-Vis spectroscopy (for organic dye) and ICP-AES (for metal ions).

3.2 Magnetic removal of microplastics

Commercially available colloidal suspension (specific gravity 1.05 g/cm^3) of polystyrene (PS) latex beads of two different sizes (1 and 10 µm diameter) were chosen as model microplastics suspensions. Both the solutions have solid PS content of 10 wt%. A dilution series of both the solutions (0.1, 0.05, 0.025 and 0.001 wt%) were prepared in distilled water and pure distilled water was used as the blank solution to build a dynamic light scattering (DLS) calibration curve of concentration and Derived Count Rate (measured in kilo-

counts per second, kcps). The 0.1 wt% solutions were used as the sample microplastics suspension. Magnetic pollutant removal experiments were conducted following the procedure described in 3.1. Subsequent removal of the magPOM-SILP by permanent magnet shows a macroscopically transparent solution, in stark contrast to the white, translucent original solutions. The results were quantified experimentally by DLS measurements. The surface coverage of the PS beads with the smaller magPOM-SILP **2** particles was demonstrated by SEM analyses (Fig Sx) and also elemental mapping, see main manuscript.

3.3 Analysis of the DLS data

Particle detection by DLS can be related back to the amount of light scattered by the sample, these values are reported as "DLS derived count rate" in kilo-counts per second, kcps. This is the observed count rate normalized to 0 % attenuation at 100 % laser power. Based on extensive analyses, this represents the most reliable particle size identification method by DLS, since DLS measurements are prone to reporting "false positive" results.[6] To ensure quantitative comparability between the measurements, all measurements were performed using identical measurement parameters (5 runs total, each comprised of 10 sub-runs; measurement duration for each sub-run was 10 seconds). Based on reference measurements using pure, microfiltered water, derived count rates (in kcps, kilo-counts per second) below 20×10^2 kcps suggest the absence of scattering centers and therefore the absence of colloidal particles. In contrast, truly colloidal reference samples give derived count rates in the range of several hundreds to thousands of kcps. The derived count rate was therefore used as a quality criterion to evaluate the presence or absence of colloids.

Table S2. Dynamic Light Scattering data for microplastic removal detection.

Solution	DLS Derived Count Rate / 10^2 kcps	Removal / %
**Pure water reference**	**10-15**	**-**
**1 µm PS beads[a]**		
**Before treatment**	**295**	**-**
**After treatment with magPOM-SILP 2**	**15**	**100**
**After treatment with reference 1**	**287**	**0**
**10 µm PS[a]**	**-**	
**Before treatment**	**1160**	**-**
**After treatment with magPOM-SILP 2**	**8**	**100**
**After treatment with reference 1**	**1150**	**0**
**Large volume removal, 10 µm PS[b]**	**-**	
$V_{solution}$ **= 20 mL**	**92.9**	**> 90 %**
$V_{solution}$ **= 50 mL**	**107.52**	**> 90 %**

[a]PS initial concentration: 0.1wt%; binding time 24 h, $V_{solution}$ = 5 mL. [b] Identical setup as [a] with the increased volumes given in the table; removal time: 6 h.

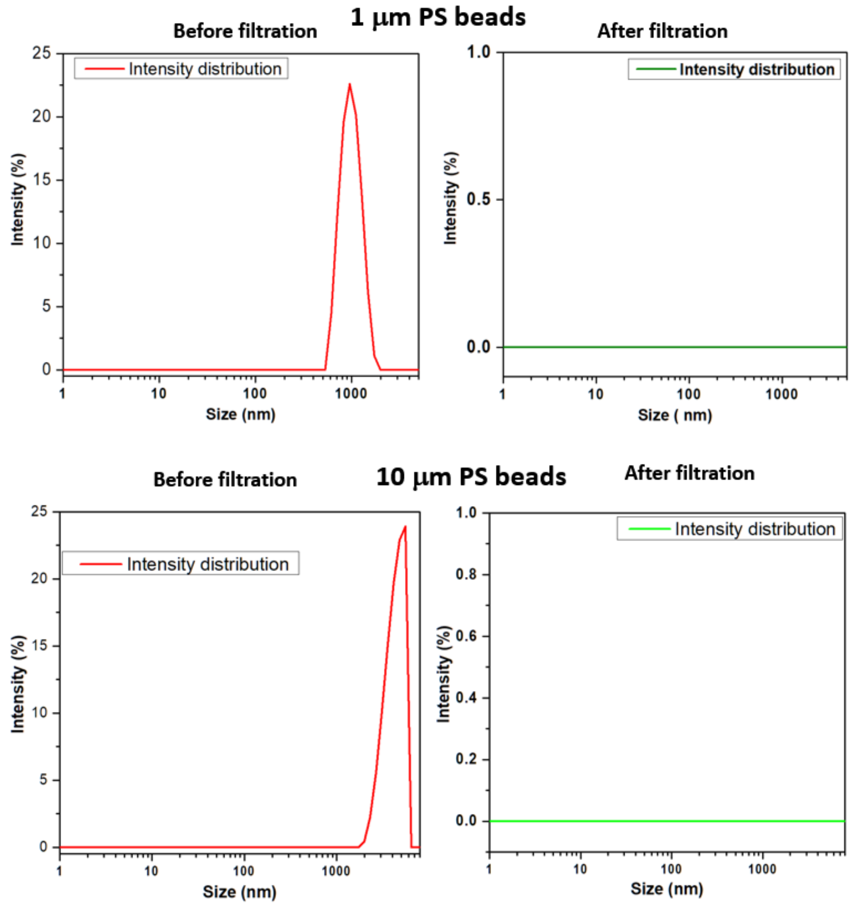

Figure S3: DLS Intensity graphs before and after removal of the PS beads (1 µm, top, 10 µm, bottom) using magPOM-SILP **2.**

Proposed microplastics removal mechanism

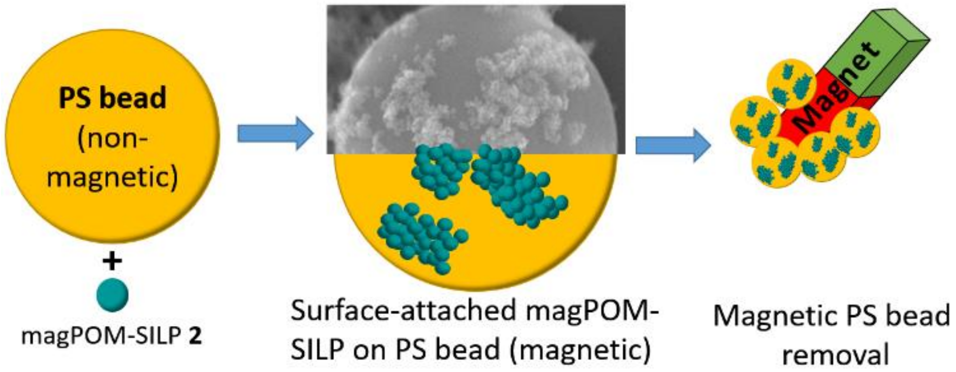

Figure S4: Proposed mechanism of microplastics removal by magPOM-SILP **2**: upon adding **2** to the PS bead colloid, surface-attachment of **2** on the larger PS bead occurs, see SEM image. We suggest this is due to hydrophobic interactions between the POM-IL on the SILP surface, and the PS bead surface. This renders the conglomerate magnetic, so that subsequent removal by a permanent magnet is possible.

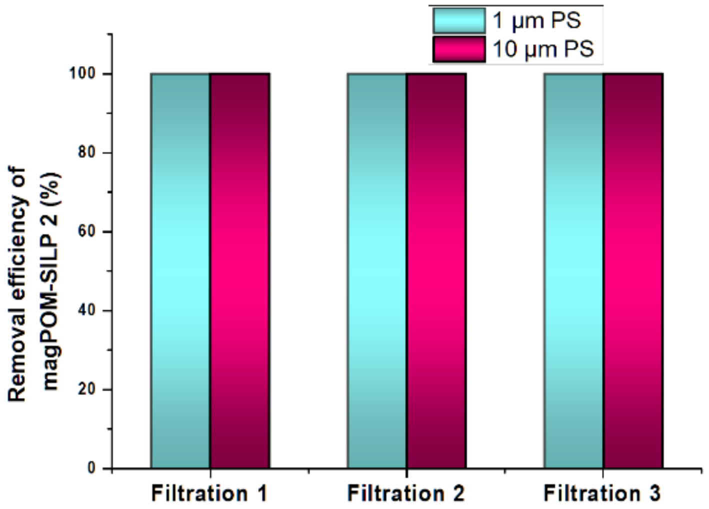

Figure S5: Recycling of the magPOM-SILP **2** after microplastics removal, showing quantitative removal of 1μm and 10 μm PS beads in three consecutive runs using standard removal conditions described above.

4. Antibacterial activity assays

4.1 Microorganisms and methods summary

All experiments were carried out under sterile conditions in a Class I Biosafety Cabinet. All data are based on six replicas to obtain average values and standard deviations and each experiment was repeated a minimum of three separate times to verify the results between different inoculations. *Escherichia coli* DH5-alpha as Gram-negative (G-) bacteria and *Bacillus subtilis* 1904-E as Gram-positive (G+) bacteria were used in the assays. Luria-Bertani (LB) liquid medium (Miller's formulation) and Nutrient Broth (NB) liquid medium were freshly prepared and sterilized by autoclave. Trypticase Soy Agar (TSA) plates were purchased from Thermo Scientific ™.

4.1.1. Bacterial disinfection protocol

Escherichia coli DH5-alpha and *Bacillus subtilis* 1904-E were pre-inoculated in Luria-Bertani (LB) medium or Nutrient Broth (NB) liquid medium, respectively, and kept under agitation (75 rpm) at 37 °C for 24 hours. A 10^6 CFU/mL inoculum of *E. coli* or *B. subtilis* was incubated with an aqueous 10 mg/mL dispersion of the magPOM-SILP in a 10 mL glass vial at 37 °C for 50 min under mild agitation. An external magnet was then used to separate the magPOM-SILP particles from the bacterial solution. The colony-forming ability (the cell viability) of *E. coli* and *B. subtilis* present in the supernatants were tested by plating serially diluted aliquots of the supernatant samples. 100 μL of each sample were plated on TSA plates and incubated overnight at 37 °C. After the incubation time, the colonies were counted and the CFU/mL of the supernatant was calculated taking into account the applied dilution. Results were compared with the optical density variation of a control culture containing only bacteria (*E. coli* or *B. subtilis*).

4.1.2. Cyclic bacterial disinfection protocol

The aforementioned protocol (4.1.1.) was followed and represented cycle 1. For subsequent cycles (2 and 3), the supernatant from cycle 1 was removed carefully using a pipette leaving only magPOM-SILPs in the glass vial, which were then washed with water. A fresh 10^6 CFU/mL inoculum of *E. coli* or *B. subtilis* was incubated with an aqueous 10 mg/mL dispersion of the magPOM-SILP in a 10 mL glass vial at 37 °C for 50 min under mild agitation. An external magnet was then used to separate 100 μL of the magPOM-SILP particles from the bacterial solution, the rest of which was discarded. The magPOM-SILP particles were washed once with water and the process was repeated for cycle 3. Colony plate counting data can be found in Table S3.

Table S3. Colony plate counting data from cyclic bacterial disinfection experiments.

CFU/mL	**Control**	**Cycle 1**	**Cycle 2**	**Cycle 3**
E. coli	4,23 x 10^5	0	0	5,00 x 10^4
B. subtilis	6,00 x 10^4	0	0	0

4.2 Sample preparation protocols used to obtain electron microscopy images

4.2.2. Scanning electron microscopy (SEM)

Bacterial cells were incubated with an aqueous 10 mg/mL dispersion of the magPOM-SILP for 50 min then centrifuged at 1400 rpm (300 G) for 10 minutes. The supernatant was removed and the pellet containing bacteria was resuspended in 1 mL of 2.5 % glutaraldehyde in phosphate buffer 10 mM pH 7.2 for fixation of the cells. The solutions were rotated for 2 hours in the Ferris wheel and afterwards the cells were washed once with 1 mL of sterile PBS and three times with sterile distilled water (centrifuged at 3000 rpm for 15 minutes between one wash and another) to remove excess glutaraldehyde. Finally, the pellets were resuspended in 200 µL of sterile MilliQ water and 2 µL of the suspension were placed on a silicon wafer. SEM images were acquired using a field emission SEM Inspect F50 (FEI Company, Eindhoven, The Netherlands) in the energy range 0–30 keV.

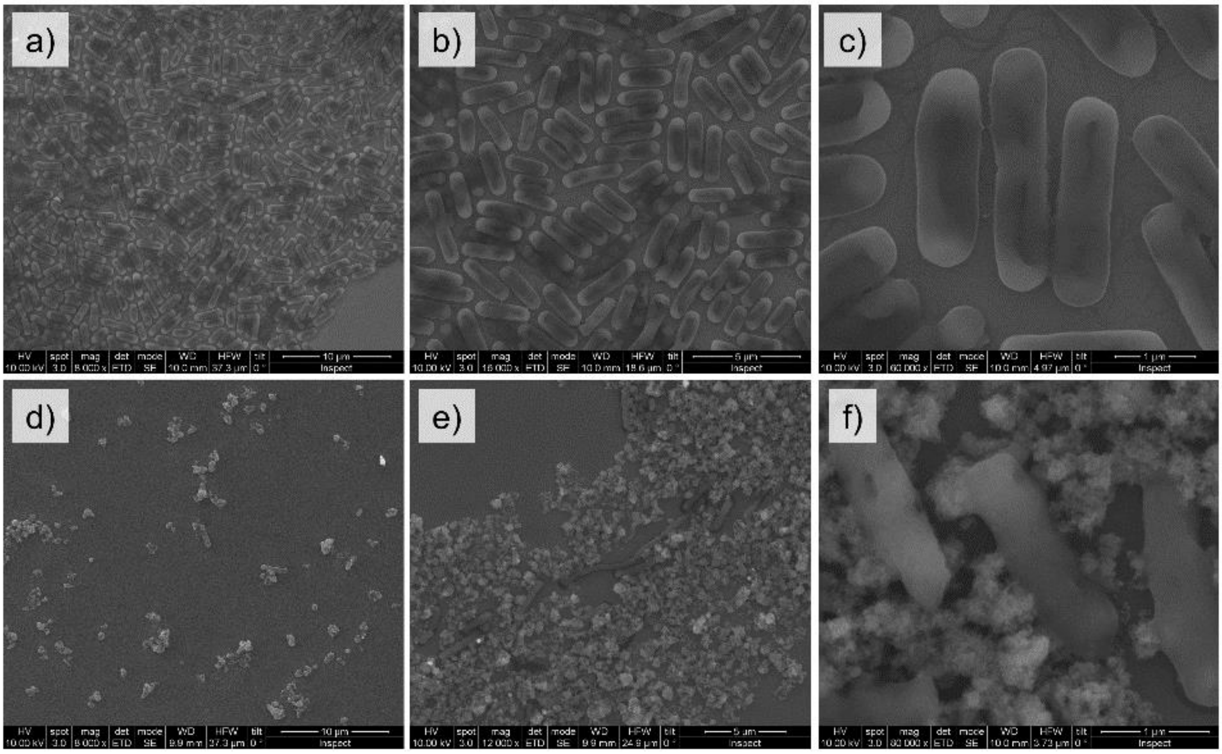

Figure S6: SEM images of a control *B. subtilis* culture without any treatment (a, b, c) and *B. subtilis* incubated with 0.5 mg/mL magPOM-SLIP **2** (d, e, f), at different magnifications.

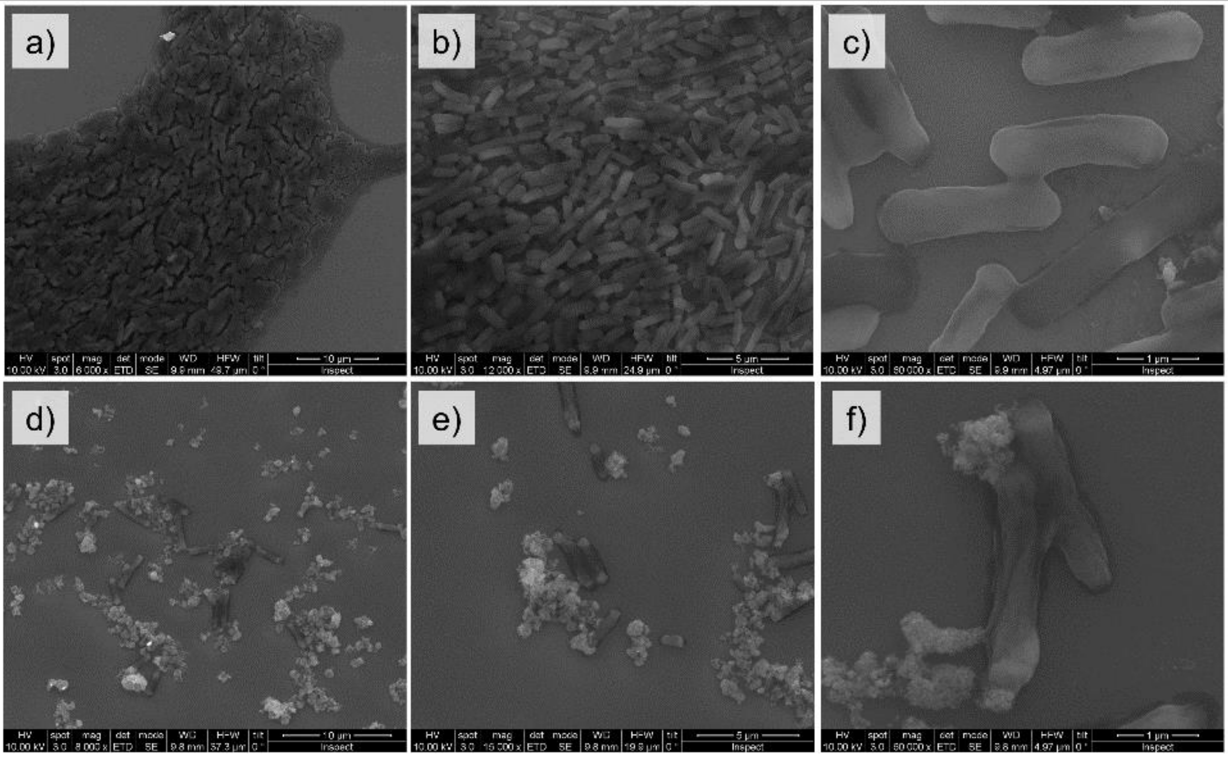

Figure S7: SEM images of a control *E. coli* culture without any treatment (a, b, c) and *E. coli* incubated with 1 mg/mL magPOM-SLIP **2** (d, e, f), at different magnifications.

4.2.1. Transmission electron microscopy (TEM)

Samples of bacteria were identical to the SEM sample preparation protocol. After the pellets were resuspended in sterile distilled water, 2 μL of the sample was deposited onto a carbon-coated copper grid (Cu200 mesh) and left to dry in air for several hours at room temperature. TEM analysis was carried out in a TECNAI T20 electron microscope (FEI) working at 60 kV.

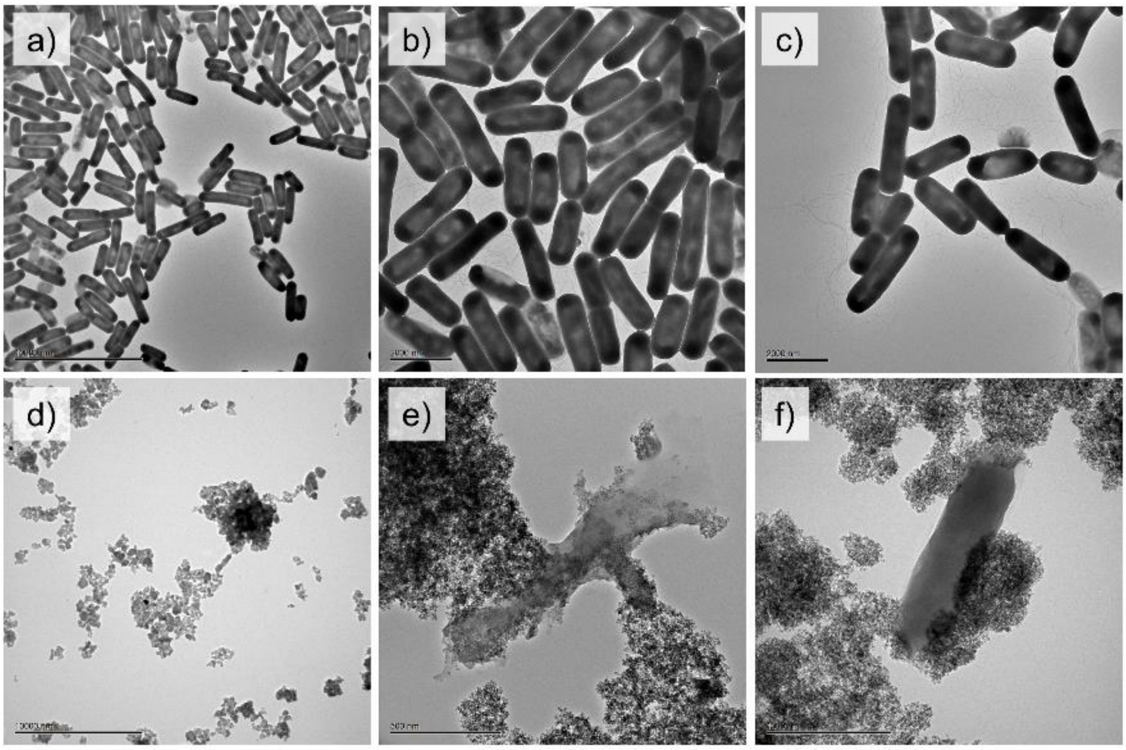

Figure S8: TEM images of a control *B. subtilis* culture without any treatment (a, b, c) and *B. subtilis* incubated with 0.5 mg/mL magPOM-SLIP **2** (d, e, f), at different magnification. In image e) the bacterial cell membrane has ruptured in presence of the particles, leaking its cytoplasmic constituents.

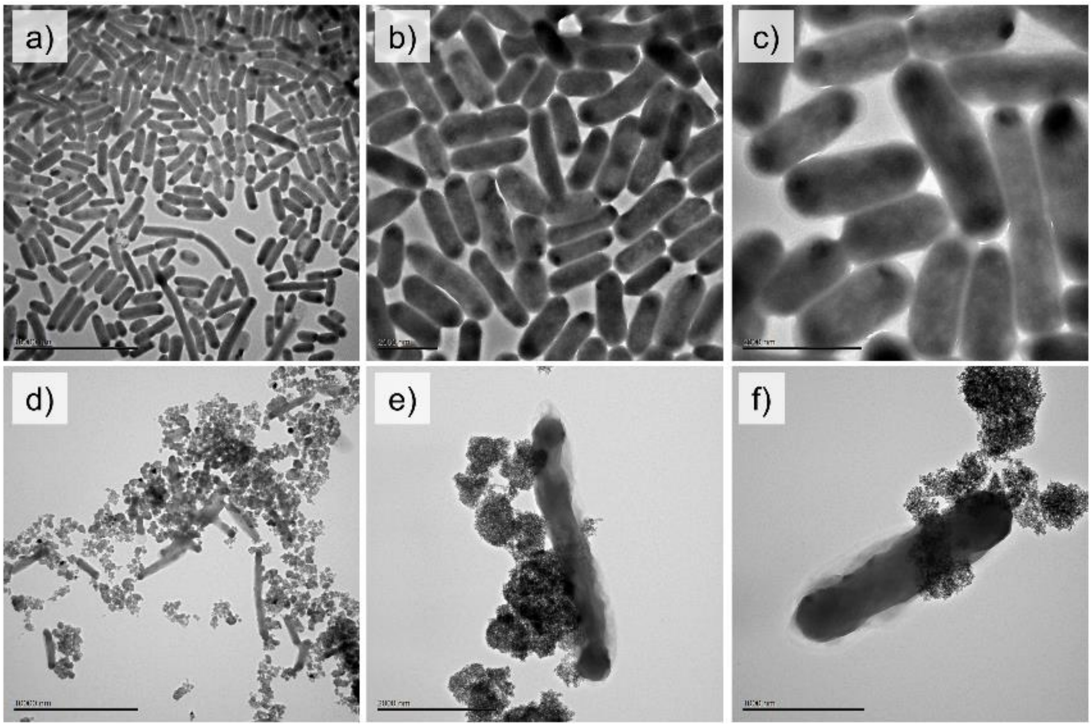

Figure S9: TEM images of a control *E. coli* culture without any treatment (a, b, c) and *E. coli* incubated with 1 mg/mL magPOM-SLIP **2** (d, e, f), at different magnifications. In the images below (d, e, f) the bacteria lose their regular shape and the cell membrane is damaged and ruptured in presence of the particles.

5. References

[1] V. W. Day, M. F. Fredrich, W. G. Klemperer, W. Shum, *J. Am. Chem. Soc.* **1977**, *99*, 6146–6148.
[2] A. Misra, I. Franco Castillo, D. P. Müller, C. González, S. Eyssautier-Chuine, A. Ziegler, J. M. de la Fuente, S. G. Mitchell, C. Streb, *Angew. Chemie Int. Ed.* **2018**, *57*, 14926–14931.
[3] S. Herrmann, M. Kostrzewa, A. Wierschem, C. Streb, *Angew. Chemie Int. Ed.* **2014**, *53*, 13596–13599.
[4] S. Herrmann, L. De Matteis, J. M. de la Fuente, S. G. Mitchell, C. Streb, *Angew. Chem. Int. Ed.* **2017**, *56*, 1667–1670.
[5] S. Takenaka, H. Umebayashi, E. Tanabe, H. Matsune, M. Kishida, *J. Catal.* **2007**, *245*, 392–400.
[6] B. Kirchhoff, S. Rau, C. Streb, *Eur. J. Inorg. Chem.* **2016**, *2016*, 1425–1429.

6. Author Contributions

A.M., C.S., R.G. and S.G.M. conceived the research and planned the experiments. A.M. and C.Z. performed materials synthesis and characterization. A.M., G.K. and A.K. performed pollutant removal studies. C.Z. and R.G. performed porosity analyses. S.G.M. and I.F.C. performed antibacterial analyses. M.H.A. performed SEM/EDX analyses. All authors co-wrote the manuscript.
